# Deep-Level Emission Tailoring in ZnO Nanostructures Grown via Hydrothermal Synthesis

**DOI:** 10.3390/nano13010058

**Published:** 2022-12-23

**Authors:** Svetlana A. Kadinskaya, Valeriy M. Kondratev, Ivan K. Kindyushov, Olga Yu. Koval, Dmitry I. Yakubovsky, Alexey Kusnetsov, Alexey I. Lihachev, Alexey V. Nashchekin, Irina Kh. Akopyan, Alexey Yu. Serov, Mariana E. Labzovskaya, Sergey V. Mikushev, Boris V. Novikov, Igor V. Shtrom, Alexey D. Bolshakov

**Affiliations:** 1 Center for Nanotechnologies, Alferov University, 194021 St. Petersburg, Russia; 2 Center for Photonics and 2D Materials, Moscow Institute of Physics and Technology, 9 Institutskiy Lane, 141701 Dolgoprudny, Russia; 3 Lab. "Characterization of Materials and Structures of Solid State Electronics", Loffe Institute, 194021 St. Petersburg, Russia; 4 Department of Solid State Physics, Saint Petersburg State University, 199034 St. Petersburg, Russia; 5 Department of Nanotechnology Methods and Instruments, IAI RAS, 198095 St. Petersburg, Russia

**Keywords:** zinc oxide, hydrothermal, nanowire, photoluminescence, deep-level emission, PEI, sodium citrate

## Abstract

Zinc oxide (ZnO) nanostructures are widely used in various fields of science and technology due to their properties and ease of fabrication. To achieve the desired characteristics for subsequent device application, it is necessary to develop growth methods allowing for control over the nanostructures’ morphology and crystallinity governing their optical and electronic properties. In this work, we grow ZnO nanostructures via hydrothermal synthesis using surfactants that significantly affect the growth kinetics. Nanostructures with geometry from nanowires to hexapods are obtained and studied with photoluminescence (PL) spectroscopy. Analysis of the photoluminescence spectra demonstrates pronounced exciton on a neutral donor UV emission in all of the samples. Changing the growth medium chemical composition affects the emission characteristics sufficiently. Apart the UV emission, nanostructures synthesized without the surfactants demonstrate deep-level emission in the visible range with a peak near 620 nm. Structures synthesized with the use of sodium citrate exhibit emission peak near 520 nm, and those with polyethylenimine do not exhibit the deep-level emission. Thus, we demonstrate the correlation between the hydrothermal growth conditions and the obtained ZnO nanostructures’ optical properties, opening up new possibilities for their precise control and application in nanophotonics, UV–Vis and white light sources.

## 1. Introduction

Zinc oxide is a technologically feasible, abundant, chemically stable, and non-toxic wide-gap semiconductor material that was actively studied during recent decades. Wide bandgap (3.37 eV at room temperature (RT) in a bulk) and large exciton binding energy (60 meV), which is much higher than thermal energy at RT, make it an excellent candidate for the development of UV light sources [1]. This material has been extensively used as a photocatalyst [2] to inactivate bacteria and viruses, and to degrade environmental pollutants such as dyes, pesticides, and volatile organic compounds with appropriate light irradiation [3,4,5].

There are various growth methods used to obtain ZnO structures for research and device applications [6]: atomic layer deposition (ALD), molecular beam epitaxy (MBE), laser deposition, etc. [7,8,9,10,11]. Semiconductor nanostructures used to develop nanoelectronic and nanophotonic devices [12,13] are most often synthesized with the epitaxial methods. Despite the fact that these methods provide numerous capabilities for control over the structures’ size and chemical composition [14,15], such synthesis is a complex technological process, expensive, and time-consuming. In addition, there are a few peculiar ZnO synthesis methods such as water oxidation [16], sputtering [17], and electrodeposition [18]. These methods, however, require sophisticated expensive equipment and, like epitaxial methods, are technologically complex.

The alternative promising method for the growth of ZnO nanostructures is hydrothermal synthesis [19]. The advantages of this include vast control over the growth conditions and low synthesis temperatures (less than 100°C) [19], providing significant reduction in energy consumption and making this technique technologically feasible. Using the hydrothermal synthesis, it is possible to obtain nanostructures of various geometries and on various substrates, both lattice-matched and not, classical ones of silicon, sapphire, and silicon carbide [8,20,21], and that are transparent and even flexible [22,23].

ZnO finds wide practical application in the form of powders [24], nanoparticles [25], films [26,27], and nanostructures [5,28,29]. Semiconductor nanostructures attract a lot of interest owing to the rapid development of the growth technologies and applications [30,31]. Nanowires (NWs) [32] have been actively studied over recent decades due to their unique physical properties [33,34]. In particular, ZnO NWs were successfully employed in development of UV lasers [35], LEDs [36], solar cells [37,38,39], and photodetectors [40]. Control over the ZnO nanostructure geometry opens possibilities for tailoring of their optical resonant properties [41] while the rich family of ZnO deep-level states [42] makes these nanostructures a versatile platform for advanced applications in photonics, optoelectronics, and sensorics [43]. The main challenge on the way to real applications is the development of growth protocols allowing for independent control over the ZnO nanostructure geometry and crystalline perfection.

Despite the wide range of works on the hydrothermal synthesis and device application of ZnO nanostructures, there have been no systematic studies aimed at simultaneously controlling the geometry of such objects and studying the growth conditions’ effect on their optical properties. In this work, samples obtained by hydrothermal synthesis with the addition of surfactants are thoroughly studied to demonstrate that change in the growth solution chemistry provides not only ability for control over the nanostructures’ morphology but also affects the luminescent properties of the structures, allowing for fine-tuning of the ZnO structures’ optical properties and their application in photonics.

## 2. Methods

### 2.1. Nanostructures’ Growth

Typically, equimolar aqueous solutions of zinc nitrate (Zn(NO_3_)_2_) and hexamethylenetetramine (HMTA–C_6_H_12_N_4_) are used for the ZnO nanostructures’ hydrothermal synthesis [19]. Here, Zn(NO_3_)_2_ serves as a source of Zn^2+^ ions, and HMTA is a slowly decomposing base that provides an alkaline environment in solution and the desired amount of OH^−^ ions. Reactions occurring during the synthesis include:

(1) $$C_{6}H_{12}N_{4}+6H_{2}O\;\to \;6\mathrm{HCHO}+4{\mathrm{NH}}_{3}$$

(2) $${\mathrm{NH}}_{3}+{\;H}_{2}O\;\to {\;\mathrm{NH}}_{4}^{+}+{\;\mathrm{OH}}^{-}$$

(3) $$\mathrm{Zn}{\left({\mathrm{NO}}_{3}\right)}_{2}\;↔{\;\mathrm{Zn}}^{2+}+2{\mathrm{NO}}_{3}$$

(4) $$2{\mathrm{OH}}^{-}+{\;\mathrm{Zn}}^{2+}\;↔\;\mathrm{Zn}{\left(\mathrm{OH}\right)}_{2}$$

(5) $$\mathrm{Zn}{\left(\mathrm{OH}\right)}_{2}\;↔\mathrm{ZnO}+{\;H}_{2}O$$

These reactions can be deviated from equilibrium by changing growth parameters such as temperature, precursor concentration, pH, etc. affecting the density of nanostructures and their aspect ratio. Another tool for controlling the nanostructures’ morphology is the addition of surfactants. Polyethyleneimine (PEI) is a polar polymer with a large number of amino groups (-NH_2_). It protonates over a wide pH range, so it is usually positively charged and tends to settle on the non-polar ZnO planes. As a result of electrostatic interaction, it suppresses the growth of nanostructures on these planes. The presence of PEI stimulates an increase in the nanostructures’ aspect ratio and, due to the suppression of the lateral growth, increases the growth time and reduces the growth species depletion rate. PEI also forms stable complexes with Zn^2+^ and does not allow the ions to precipitate.

Another surfactant for controlling the ZnO structures’ geometry is sodium citrate. Citrate ions are negatively charged, therefore, unlike PEI, they selectively bind to Zn^2+^ ions on the (0001) surface, block growth along the c-axis, and stimulate it along [01$\bar{1}$0] and [21$\bar{1}$0] directions. This process leads to the pronounced lateral extension and decay of the structures’ aspect ratio, leading to formation of hexagonal nano- and micro-sized prisms. It should be noted that the addition of sodium citrate at high concentrations (≥10 mmol∙L^−1^) inhibits growth in all directions followed by the formation of spherical particles [19].

In this work, we employ a conventional synthesis protocol using equimolar concentration Zn(NO_3_)_2_ and HMTA aqueous solutions [19]. Synthesis is carried out on Si (111) substrates, which were preliminarily purified in acetone and then in isopropanol. Five samples were synthesized: Samples 1–3 without the use of surfactants, Sample 4 with sodium citrate, and Sample 5 with PEI. For ZnO surface nucleation, we spin-coated the substrates with 3 seed layers of zinc acetate aqueous solution at a concentration of 5 mmol∙L^−1^ [44].

For the growth, HMTA aqueous solution (with the surfactants for Samples 4 and 5) was added to the zinc nitrate solution in a 200 mL Teflon cup with constant stirring. To study the influence of the solution chemical composition on the geometry of the synthesized nanostructures in the first growth series (hereinafter referred to as series A, without the surfactants), the concentrations of precursors were varied: Sample 1—50 mmol∙L^−1^, Sample 2—100 mmol∙L^−1^, Sample 3—300 mmol∙L^−1^. Samples with surfactants (4, 5, hereinafter referred to as series B) were grown with equimolar concentration of precursors of 100 mmol∙L^−1^. During the synthesis, a constant temperature of 85 °C was maintained. The synthesis duration for all samples was 3 h.

### 2.2. Raman Spectroscopy

To study structure and composition of the synthesized samples, Raman spectra were obtained of the reflection geometry at an excitation wavelength of 532 nm (2.33 eV) at a room temperature of 300 K on a Jobin Yvon Horiba LabRAM HR 800 spectrometer (Horiba Jobin Yvon, Paris, France ) equipped with an Olympus IX71 optical microscope (Olympus Corporation, Shinjuku, Tokyo, Japan) with a 100-fold magnification objective that allowed focusing of the beam onto a ~1 μm spot and provided excitation power of 3.94 mW.

### 2.3. Grazing Incidence X-ray Diffraction (GIXRD) Analysis

In order to check the crystallinity and confirm purity of the samples, GIXRD analysis was performed. Powder GIXRD data were obtained on a Thermo ARL X’TRA H-8 (Thermo Fisher Scientific, Waltham, MA, USA) diffractometer, and the radiation was generated by an IμS micro-focus X-ray tube with Cu-Kα radiation (λ = 1.5418 Å, ~8 keV). XRD data were analyzed using the ILL Grenoble FullProf Version (June 2022), (Winplotr) software package [45].

### 2.4. Photoluminescence Spectroscopy

Low-temperature photoluminescence (PL) spectra provide important information about processes involving electrons, holes, excitons, donors, and acceptors. In particular, using PL spectroscopy the role of sample growth method (ALD, MBE, laser deposition), laser effects, desorption and adsorption processes, and induced effects [46,47,48,49,50,51] was studied previously. The PL spectra of the synthesized samples were studied using an MDR-204-2 monochromator (LOMO-Photonics, St. Petersburg, Russia). The samples were placed in a closed-cycle helium cryostat (Janis Research Company, Woburn, MA, USA). The PL was excited by a He-Cd laser (excitation wavelength λ = 325 nm, maximum radiation power W = 50 kW∙cm^−2^). The sample temperature was varied in the 5–300 K range. The laser radiation intensity was controlled by neutral light filters.

## 3. Results and Discussion

### 3.1. Nanostructures’ Morphology

**Figure 1:**
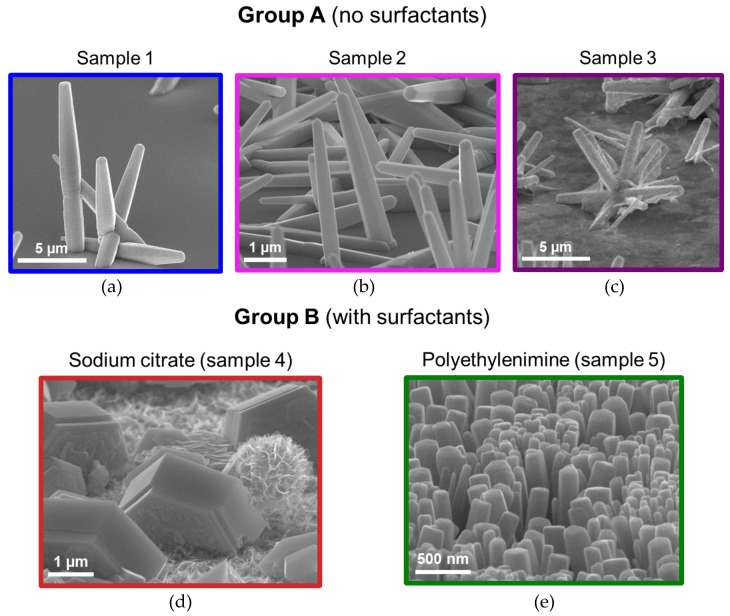
SEM images of the synthesized samples: (**a**) grown without surfactants, precursor concentration 50 mmol∙L^−1^; (**b**) no surfactants, concentration of precursors 100 mmol∙L^−1^; (**c**) no surfactants, concentration of precursors 300 mmol∙L^−1^; (**d**) grown with sodium citrate, precursor concentration 100 mmol∙L^−1^; (**e**) synthesis using PEI, precursor concentration 100 mmol∙L^−1^.

Typical images of the synthesized ZnO nanostructures obtained using a JSM 7001F scanning electron microscope (SEM) (JEOL, Akishima, Tokyo, Japan) are shown in Figure 1. According to the analysis of the images, NWs and microcrystals with hexagonal faceting were obtained. The first two samples of series A (without surfactants, Figure 1a,b) exhibit misoriented NWs with an aspect ratio around 10:1. NW surface density in Sample 2 is ~5-fold higher than in Sample 1. We assume that such a phenomenon is the result of an increase in the precursors’ content in the growth solution in Sample 2. However, length of a single nanostructure decreases in this sample compared to Sample 1, which is probably due to the competition of the denser nanostructures for the growth species. Sample 3 exhibits complex morphology structures consisting of misoriented NWs each having the geometry typical of the previous experiments. 

Sodium citrate, as expected, suppressed the growth along the c-axis, leading to lateral outgrowth and formation of misoriented hexagonal microcrystals in Sample 4 with a diameter of ~7 µm and a height of ~ 2–3 µm (Figure 1d). Such a geometry is promising for photonics and optoelectronics due to the resonant optical properties [52]. Indeed, whispering gallery modes of these structures are governed by their sizes and can be controlled by changing the growth parameters (e.g., time), while emission efficiency is the product of the structures’ surface density and can be controlled with the seed layer and precursor content.

When PEI is added to the growth solution (Sample 5), a dense array of vertically oriented NWs with a diameter of ~100 nm and a height of ~1 μm grows on the substrate surface (see Figure 1e). 

### 3.2. Raman Spectroscopy

**Figure 2:**
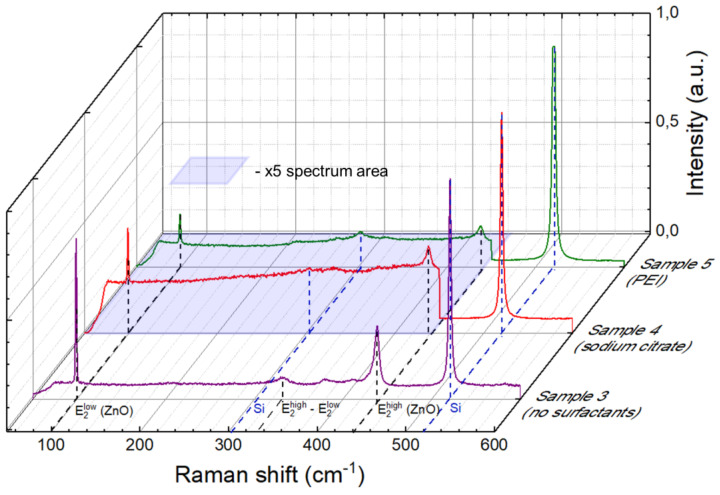
Raman spectra of: Sample 3 without surfactants (violet spectrum), Sample 4 using sodium citrate (red spectrum), Sample 5 using PEI (green spectrum). The signal intensity of Samples 4 and 5 was increased five-fold in the region of 50–440 cm^−1^ for visibility.

Three samples were selected for this study corresponding to different growth chemistry: without surfactants (Sample 3), with sodium citrate (Sample 4), and using PEI (Sample 5). Figure 2 shows typical Raman spectra for the three selected samples. The most intense Raman band in all the obtained spectra located at ~520 cm^−1^ corresponds to the fundamental silicon substrate vibrational (TO-LO) Raman mode [53]. In addition to this peak, samples also show a response from the substrate near 304 cm^−1^, which can be attributed to the acoustic (TA-LA) Raman band at the edge of the Brillouin zone. 

As for the grown nanostructures’ Raman response, the spectra exhibit ZnO vibrational modes at 99 cm^−1^ ($E_{2}^{L}$) and 439 cm^−1^ ($E_{2}^{H}$) in the center of the Brillouin zone (Γ valley) [54,55]. Occurrence of these characteristic peaks is a clear manifestation of the crystalline ZnO nanostructures’ formation. To enhance visual appeal, intensity of the Raman signal of Samples 4 and 5 was increased by a factor of 5 in the 50–440 cm^−1^ range in Figure 2. 

Sample 3 demonstrates the most intense Raman response, which is probably caused by the largest amount of material in the laser spot area. In addition to the two ZnO fundamental modes discussed above, this sample also exhibits a vibrational mode at 333 cm^−1^, which can be attributed to the $E_{2}^{H}$–$E_{2}^{L}$ (Γ) multiphonon mode at the center of the Brillouin zone [55,56].

### 3.3. Grazing Incidence X-ray Diffraction (GIXRD) Analysis

**Figure 3:**
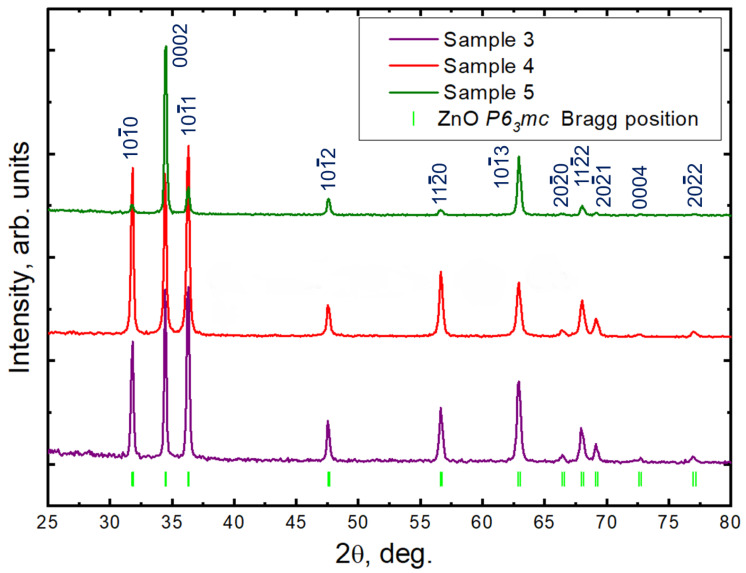
Normalized over the maximum intensity value of powder GIXRD intensity patterns of the studied samples. The patterns are shifted vertically to enhance the visual appeal.

The GIXRD patterns of the as synthesized ZnO/Si(111) structures are presented in Figure 3 and show well-defined diffraction peaks of the ZnO *P6_3_mc* wurtzite crystal phase (JCPDS Data Card No: 36-1451) [57,58,59]. Structures are found free of impurities as they do not exhibit any characteristic XRD peaks other than that corresponding to ZnO. As clearly seen in Figure 3, all of the Bragg reflexes are bright and sharp, manifesting the good crystalline quality of the synthesized structures [59]. The absence of a wide plateau and dramatic broadening of the reflexes in the XRD patterns proves the absence of amorphous ZnO [60,61].

The *a* and *c* lattice constants are refined using the Rietveld method [62,63] and demonstrate excellent agreement with the lattice parameters of nanostructured ZnO materials reported previously (*a*—3.2504 Å and *c*—5.2063 Å, respectively) [57] and differ from the values corresponding to the monocrystal bulk (JCPDS Data Card No: 36-1451).

Deviant behavior of the reflex intensity in the XRD patterns is the evidence of a difference in the lattice orientation in the synthesized samples. For example, in the GIXRD pattern of Sample 5, reflex 0002 has a maximum intensity, that can be described by the growth of large and dense hexagonal structures oriented along the ZnO c-axis on Si(111) substrate and correlates with the SEM image analysis. That indicates the effective suppression of four equivalent growth directions due to the use of seed layers and optimal conditions of the hydrothermal synthesis. This phenomenon was also observed previously [64,65,66,67].

### 3.4. Photoluminescence Spectroscopy

Photoluminescence spectroscopy was used to study the growth conditions’ influence on the optical properties of the synthesized ZnO nanostructures. First, all the samples were characterized under cryogenic conditions in a narrow spectral range near the absorption edge. Noteworthily, to make the study straightforward we excited the samples in different spots. The PL intensity was found to vary slightly from point to point, but the PL spectral features remained.

**Figure 4:**
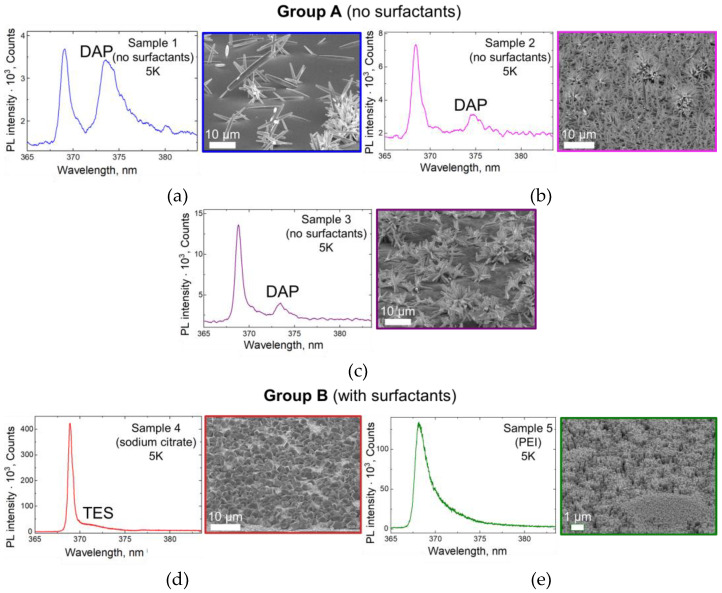
PL spectra taken at T = 5 K and corresponding SEM images of the samples: (**a**) Sample 1, (**b**) Sample 2, (**c**) Sample 3, (**d**) Sample 4, (**e**) Sample 5.

Figure 4a–c show the characteristic PL spectra of the group A samples, taken under the same conditions. It can be seen that the near-band-edge (NBE) PL of all the samples in this group at T = 5 K consists of two emission bands—a narrow band with a maximum at λ = 368.4–369.1 nm and a wider one centered at λ = 373.4–374.6 nm—which we call hereafter shortwave and longwave, respectively. According to the literature, the shortwave band belongs to an exciton bound to a neutral donor (D_0_X). 

Discussing the correlation between the samples’ PL spectra and their growth conditions, it is important to note that as the precursors’ content in group A changes (from 50 mmol∙L^−1^ in Sample 1 to 300 mmol∙L^−1^ in Sample 3), the overall PL intensity increases. In addition, the ratio of the shortwave and longwave bands’ intensities in these samples is different: in Sample 1, the bands have approximately the same intensity, while in Samples 2 and 3 the shortwave band dominates.

The spectral position of the shortwave emission band peak slightly changes from sample to sample in the range from 368.4 nm to 369.1 nm. Here, we note that ZnO is well-known for extensive PL peculiarities with possibly more than 10 exciton lines bound on neutral donors in a narrow spectral range, which can be, for example, attributed to H, Al, In, Ga [68], and intrinsic defects of ZnO, which will be discussed hereafter. The observed line at λ = 368.6 nm in the literature is usually associated with the presence of hydrogen atoms. The shorter wavelength line in these samples (λ= 368.4 nm) is possibly related to the exciton localized on the surface centers of the acceptor [68].

The broader long-wavelength emission band in the spectra of the group A samples (λ = 373.4–374.6) has a complex nature and is usually associated with the transition of donor–acceptor pairs (DAPs) [69,70], which affects its shape and spectral position. Impurities and intrinsic defects can act as acceptors here. For example, to develop p-type ZnO samples, they are usually doped with nitrogen, which leads to a strong increase in this band intensity. Thus, the use of the nitrogen-containing Zn(NO_3_)_2_ can affect the manifestation of this band.

The PL spectra of groups A and B have a noticeable difference. The emission intensity of the group B samples is an order stronger than that of group A (see Figure 4). In the PL spectra of the group B samples, only one narrow band is detected: λ = 368.6 nm, whose position coincides with the D_0_X band in group A PL spectra. In Sample 4, a “shoulder” appears at 372 nm, most likely due to a two-electron satellite transition (TES) [69,70]. The higher PL intensity and the absence of a pronounced DAP band are most likely associated with the higher crystaline perfection of the group B samples.

### 3.5. Deep-Level Emission and PL Temperature Dependence

**Figure 5:**
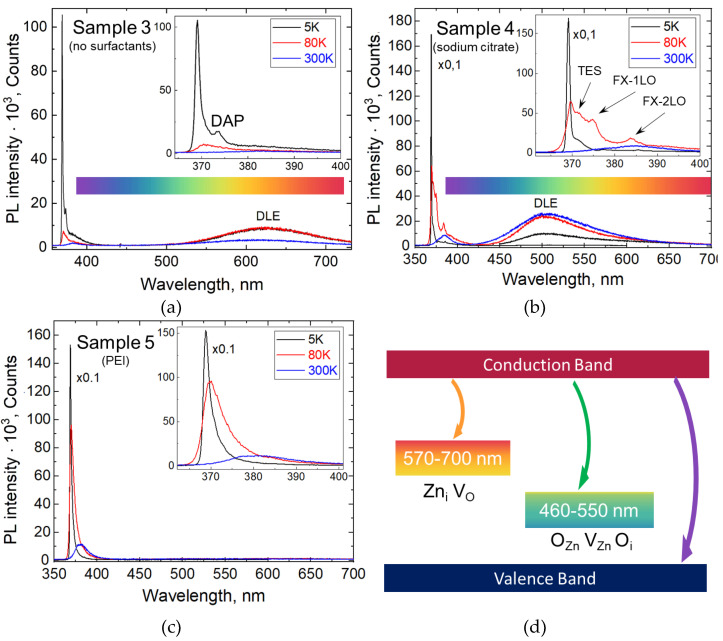
PL spectra taken in a wide spectral range at different temperatures: (**a**) Sample 3 (without surfactant), (**b**) Sample 4 (with sodium citrate), NBE region of the spectrum is multiplied by 0.1, (**c**) Sample 5 (with PEI) NBE region of the spectrum multiplied by 0.1. Insets (**a**–**c**): enlarged short-wavelength region of the PL spectra. (**d**) Schematic representation of the deep levels and corresponding radiative transitions.

ZnO structures are well known for the wide range of deep defect levels, allowing radiative recombination with emission bands in the visible range known as deep-level emission (DLE) [42]. To study the growth conditions’ influence on these bands’ manifestation, we carried out a wide spectral range PL study. For this, we selected Sample 3 from group A, exhibiting the highest PL intensity, and both samples from group B due to the different surfactants used in their synthesis. PL spectra were taken in the 350–700 nm range covering the whole visible range at three temperatures—5 K, 80 K and 300 K—and they are shown in Figure 5.

The richest fine structure of the UV emission is observed in Sample 4 at a temperature of 80 K. It consists of four bands lying in the range from 370 nm to 383.6 nm with the most pronounced corresponding to the D_0_X. The "shoulder" at 372 nm is related to TES [69,70] while the peaks at 375 nm and 383.6 nm are attributed to the first (1LO) and second order longitudinal optical (2LO) phonon replica of the free exciton (FX) emission according to previous results [71]. 

In addition to the UV NBE emission, Sample 3 exhibits a DLE band in the visible range centered near 620 nm. This band is often associated with excess zinc including Zn interstitial (Zn_in_) and lack of oxygen such as vacancies (V_O_) [42,72,73], which may be due to the high concentration of precursors in the growth solution and, consequently, the high content of Zn^2+^ ions. Corresponding schematics of the radiative transition of an electron from the conduction band to the deep levels related to the excess zinc and oxygen vacancies are shown in Figure 5d in orange.

Sample 4 also exhibits DLE. In this case, the emission band is centered near 520 nm. This behavior is associated with the excess oxygen and zinc vacancies [42,72,73,74]. We note that Sample 4 was synthesized with sodium citrate. We believe that addition of this surfactant leads to a shift in the ion balance in the growth solution due to the partial capture of Zn ions by negatively charged citrate groups. As such, formation of ZnO microcrystals takes place in a shortage of Zn atoms. Corresponding schematics of the radiative transition of an electron from the conduction band to the deep levels related to excess oxygen (O_i_ interstitials and O_Zn_ substitutions) and zinc vacancies (V_Zn_) are shown in Figure 5d in green.

Unlike the previous structures, Sample 5 does not exhibit DLE, which is a non-trivial result [75,76,77]. We note that the use of PEI as a surfactant suppresses the lateral ZnO crystals’ growth. Thus, the growth occurs due to the species attachment to the small polar facets. We believe that limitation of the growth facet size leads to a slow zinc and oxygen local material consumption, and the growth becomes materially balanced, leading to the high crystalline perfection of the grown nanostructures.

## 4. Conclusions

In this work, high crystallinity ZnO nanostructures of various geometries are synthesized using a hydrothermal method with the addition of surfactants. 

PL spectroscopy study demonstrates strong NBE emission in the UV region in all of the synthesized samples associated with an exciton on a neutral donor (D_0_X). The obtained spectra demonstrate the different responses in the visible range governed by the deep levels. The sample synthesized without the surfactants has an efficient response in the visible range, centered near 620 nm. Use of sodium citrate leads to the DLE in the green region centered near 500 nm, while use of PEI makes it possible to suppress the DLE. These effects are associated with a variation in the balance between zinc and oxygen ions in the growth solution provided by the change in the chemical composition of the growth medium.

The obtained results demonstrate the prospects for use of the technologically feasible hydrothermal method to develop light-emitting structures based on zinc oxide, which can compete with organic light-emitting diodes (OLEDs), phosphorescent organic light-emitting diodes (PHOLEDs), and white organic light-emitting diodes (WOLEDs) [78,79] in the future. The spectral characteristics of such structures can be tailored in a wide range by changing the growth medium composition, opening the way for fabrication of UV–Vis and white light sources for biology, disinfection and lighting.

## Data Availability

The data presented in this study are available on request from the corresponding author.
